# 11,12-Dichloro­dibenzo[*a*,*c*]phenazine

**DOI:** 10.1107/S1600536812049690

**Published:** 2012-12-08

**Authors:** Arta Imeri, Neil M. Glagovich, Guy Crundwell

**Affiliations:** a Department of Chemistry, Central Connecticut State University, New Britain, CT 06053, USA

## Abstract

The title compound, C_20_H_10_Cl_2_N_2_, has crystallographic twofold rotational symmetry [maximum deviation from the least-squares plane = 0.038 (1) Å]. In the crystal, weak π–π ring stacking inter­actions occur down the *a-*axis direction [minimum centroid–centroid separation = 3.7163 (8) Å].

## Related literature

For the synthesis of the title compound, see: Bellizzi *et al.* (2006 ▶). For the structures of similar compounds, see: Bellizzi *et al.* (2006 ▶); Day *et al.* (2002 ▶); Richards *et al.* (2009 ▶). 
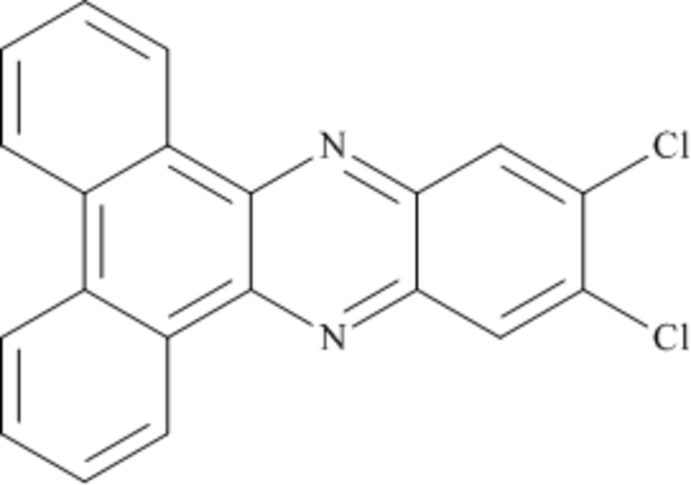


## Experimental

### 

#### Crystal data

C_20_H_10_Cl_2_N_2_
*M* *_r_* = 349.20
Monoclinic, 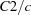

*a* = 3.8583 (3) Å
*b* = 26.2739 (13) Å
*c* = 15.1147 (10) Å
β = 94.877 (6)°
*V* = 1526.67 (17) Å^3^
*Z* = 4
Mo *K*α radiation
μ = 0.43 mm^−1^
*T* = 293 K
0.32 × 0.14 × 0.09 mm

#### Data collection

Oxford Diffraction Xcalibur Sapphire3 CCD diffractometer
Absorption correction: multi-scan (*CrysAlis PRO*; Oxford Diffraction, 2009 ▶) *T*_min_ = 0.915, *T*_max_ = 1.000
5082 measured reflections
2520 independent reflections
1372 reflections with *I* > 2σ(*I*)
*R*_int_ = 0.020

#### Refinement

*R*[*F* ^2^ > 2σ(*F* ^2^)] = 0.039
*wR*(*F* ^2^) = 0.104
*S* = 0.83
2520 reflections
109 parameters
H-atom parameters constrained
Δρ_max_ = 0.26 e Å^−3^
Δρ_min_ = −0.25 e Å^−3^

### 

Data collection: *CrysAlis CCD* (Oxford Diffraction, 2009 ▶); cell refinement: *CrysAlis RED* (Oxford Diffraction, 2009 ▶); data reduction: *CrysAlis RED*; program(s) used to solve structure: *SHELXS97* (Sheldrick, 2008 ▶); program(s) used to refine structure: *SHELXL97* (Sheldrick, 2008 ▶); molecular graphics: *PLATON* (Spek, 2009 ▶); software used to prepare material for publication: *SHELXTL* (Sheldrick, 2008 ▶).

## Supplementary Material

Crystal structure: contains datablock(s) global, I. DOI: 10.1107/S1600536812049690/zs2242sup1.cif

Structure factors: contains datablock(s) I. DOI: 10.1107/S1600536812049690/zs2242Isup2.hkl

Supplementary material file. DOI: 10.1107/S1600536812049690/zs2242Isup3.cml

Additional supplementary materials:  crystallographic information; 3D view; checkCIF report

## Figures and Tables

**Fig. 1.**
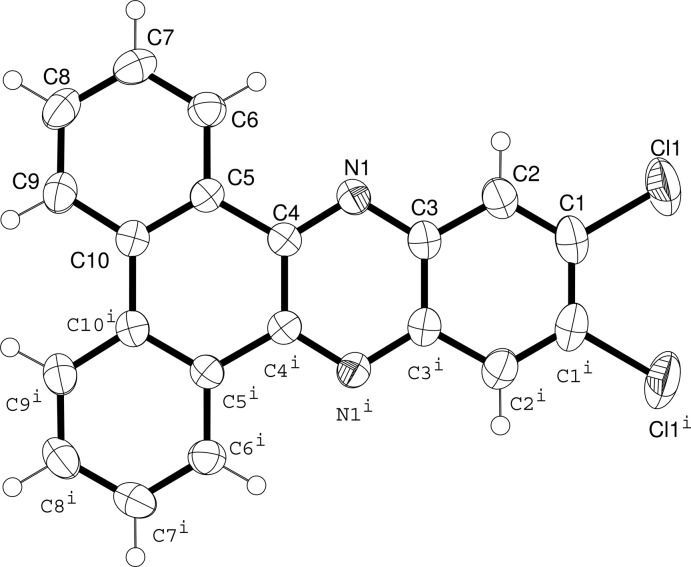
A view of the title compound showing atom numbering, with displacement ellipsoids drawn at the 50% probability level. Symmetry code (i) (-x, y, -z + 1/2), indicates atoms related by twofold rotational symmetry.

## supplementary crystallographic information

### Comment

The title compound C_20_H_10_Cl_2_N_2_ is planar [maximum deviation from the l.s. plane over the 24 atoms = 0.038 (1) Å] and lies on a crystallographic twofold axis (Fig. 1). All bond lengths and angles fall within typical ranges found in other dibenzo[a,c]phenazines (Day *et al.*, 2002; Richards *et al.*, 2009). In the crystal, the molecules are involved in weak π–π-stacking interactions [minimum ring centroid separation = 3.7163 (8) Å], giving stacks down the *a* axial direction of the unit cell.

### Experimental

To a 25 ml round bottom flask equipped with a reflux condenser was added 0.27 g (1.5 mmol) of 4,5-dichloro-1,2-phenylenediamine, 0.27 g (1.3 mmol) of phenanthraquinone, and 10 ml of glacial acetic acid (Bellizzi *et al.*, 2006) and the mixture was heated with refluxing for 6 h. After this time, the resulting yellow solution was concentrated under reduced pressure, and the product obtained was purified by recrystallization from methanol, producing 0.48 g of the title compound as a yellow solid (m.p. 273 °C; yield: 92%). R*f* 0.31 (SiO_2_, 80% hexanes-ethyl acetate); ^1^H NMR (300 MHz, CDCl_3_) d 9.35 (dd, 2H, *J* = 8.0 Hz, *J* = 1.5 Hz), 8.58 (d, 2H, *J* = 8.0 Hz, *J* = 1.2 Hz), 8.47 (s, 2H), 7.86 (dt, 2H, *J* = 7.2 Hz, *J* = 1.5 Hz), 7.774 (dt, 2H, *J* =7.2 Hz, *J* = 1.2 Hz); ^13^C NMR (300 MHz, CDCl_3_) d 143.30, 140.76, 134.30, 132.32, 130.92, 129.85, 129.79, 128.16, 126.45, 123.03; UV/Vis (CH_2_Cl_2_; λ_max_) 260 nm.

### Refinement

Hydrogen atoms were included in calculated positions with a C—H distance of 0.93 Å in the refinement in a riding motion approximation, with *U*_iso_ = 1.2*U*_eq_ of the carrier atom.

### Crystal data

**Table:**

C_20_H_10_Cl_2_N_2_	*F*(000) = 712
*M**_r_* = 349.20	*D*_x_ = 1.519 Mg m^−^^3^
Monoclinic, *C*2/*c*	Melting point: 546 K
Hall symbol: -C 2yc	Mo *K*α radiation, λ = 0.71073 Å
*a* = 3.8583 (3) Å	Cell parameters from 1714 reflections
*b* = 26.2739 (13) Å	θ = 4.1–32.5°
*c* = 15.1147 (10) Å	µ = 0.43 mm^−^^1^
β = 94.877 (6)°	*T* = 293 K
*V* = 1526.67 (17) Å^3^	Needle, yellow
*Z* = 4	0.32 × 0.14 × 0.09 mm

### Data collection

**Table:**

Oxford Diffraction Xcalibur Sapphire3 CCD diffractometer	2520 independent reflections
Radiation source: Enhance (Mo) X-ray Source	1372 reflections with *I* > 2σ(*I*)
Graphite monochromator	*R*_int_ = 0.020
Detector resolution: 16.1790 pixels mm^-1^	θ_max_ = 32.6°, θ_min_ = 4.1°
ω scans	*h* = −5→5
Absorption correction: multi-scan (*CrysAlis PRO*; Oxford Diffraction, 2009)	*k* = −27→38
*T*_min_ = 0.915, *T*_max_ = 1.000	*l* = −21→10
5082 measured reflections	

### Refinement

**Table:**

Refinement on *F*^2^	Primary atom site location: structure-invariant direct methods
Least-squares matrix: full	Secondary atom site location: difference Fourier map
*R*[*F*^2^ > 2σ(*F*^2^)] = 0.039	Hydrogen site location: inferred from neighbouring sites
*wR*(*F*^2^) = 0.104	H-atom parameters constrained
*S* = 0.83	*w* = 1/[σ^2^(*F*_o_^2^) + (0.0575*P*)^2^] where *P* = (*F*_o_^2^ + 2*F*_c_^2^)/3
2520 reflections	(Δ/σ)_max_ = 0.001
109 parameters	Δρ_max_ = 0.26 e Å^−^^3^
0 restraints	Δρ_min_ = −0.25 e Å^−^^3^

### Special details

**Table:**

Experimental. Hydrogen atoms were included in calculated positions with a C—H distance of 0.93 Å and were included in the refinement in riding motion approximation with *U*_iso_ = 1.2*U*_eq_ of the carrier atom.
Geometry. All e.s.d.'s (except the e.s.d. in the dihedral angle between two l.s. planes) are estimated using the full covariance matrix. The cell e.s.d.'s are taken into account individually in the estimation of e.s.d.'s in distances, angles and torsion angles; correlations between e.s.d.'s in cell parameters are only used when they are defined by crystal symmetry. An approximate (isotropic) treatment of cell e.s.d.'s is used for estimating e.s.d.'s involving l.s. planes.
Refinement. Refinement of *F*^2^ against ALL reflections. The weighted *R*-factor *wR* and goodness of fit *S* are based on *F*^2^, conventional *R*-factors *R* are based on *F*, with *F* set to zero for negative *F*^2^. The threshold expression of *F*^2^ > σ(*F*^2^) is used only for calculating *R*-factors(gt) *etc*. and is not relevant to the choice of reflections for refinement. *R*-factors based on *F*^2^ are statistically about twice as large as those based on *F*, and *R*- factors based on ALL data will be even larger.

### Fractional atomic coordinates and isotropic or equivalent isotropic displacement parameters (Å^2^)

**Table:**

	*x*	*y*	*z*	*U*_iso_*/*U*_eq_	
Cl1	0.13563 (12)	1.243825 (14)	0.15408 (3)	0.07256 (18)	
C1	0.0618 (3)	1.18700 (5)	0.20681 (10)	0.0463 (3)	
C2	0.1244 (3)	1.14234 (5)	0.16590 (9)	0.0430 (3)	
H2	0.2072	1.1427	0.1099	0.052*	
C3	0.0651 (3)	1.09527 (4)	0.20751 (8)	0.0355 (3)	
N1	0.1324 (3)	1.05122 (4)	0.16541 (7)	0.0356 (2)	
C4	0.0669 (3)	1.00812 (4)	0.20704 (8)	0.0308 (3)	
C5	0.1338 (3)	0.95984 (4)	0.16356 (8)	0.0306 (3)	
C6	0.2605 (3)	0.95979 (5)	0.07952 (8)	0.0390 (3)	
H6	0.3017	0.9906	0.0519	0.047*	
C7	0.3246 (4)	0.91492 (5)	0.03731 (9)	0.0447 (3)	
H7	0.4078	0.9152	−0.0187	0.054*	
C8	0.2642 (4)	0.86907 (5)	0.07896 (9)	0.0472 (4)	
H8	0.3089	0.8386	0.0508	0.057*	
C9	0.1394 (3)	0.86825 (5)	0.16127 (9)	0.0421 (3)	
H9	0.1010	0.8371	0.1880	0.051*	
C10	0.0685 (3)	0.91342 (4)	0.20592 (8)	0.0321 (3)	

### Atomic displacement parameters (Å^2^)

**Table:**

	*U*^11^	*U*^22^	*U*^33^	*U*^12^	*U*^13^	*U*^23^
Cl1	0.0829 (3)	0.0336 (2)	0.1023 (4)	−0.00414 (17)	0.0140 (3)	0.0163 (2)
C1	0.0413 (8)	0.0305 (6)	0.0665 (9)	−0.0025 (5)	0.0004 (7)	0.0067 (6)
C2	0.0440 (8)	0.0364 (7)	0.0490 (8)	−0.0003 (6)	0.0053 (6)	0.0062 (6)
C3	0.0333 (7)	0.0311 (6)	0.0417 (6)	−0.0006 (5)	0.0010 (5)	0.0014 (5)
N1	0.0380 (6)	0.0323 (5)	0.0366 (5)	0.0000 (4)	0.0047 (5)	0.0029 (4)
C4	0.0281 (7)	0.0325 (6)	0.0315 (6)	0.0005 (4)	0.0014 (5)	0.0018 (5)
C5	0.0272 (6)	0.0327 (6)	0.0315 (6)	0.0025 (5)	0.0006 (5)	−0.0012 (5)
C6	0.0393 (7)	0.0410 (7)	0.0375 (7)	0.0028 (5)	0.0074 (6)	0.0012 (5)
C7	0.0450 (8)	0.0526 (8)	0.0373 (7)	0.0081 (6)	0.0072 (6)	−0.0067 (6)
C8	0.0549 (9)	0.0403 (7)	0.0464 (8)	0.0090 (6)	0.0041 (6)	−0.0115 (6)
C9	0.0476 (8)	0.0317 (6)	0.0467 (8)	0.0028 (5)	0.0018 (6)	−0.0027 (5)
C10	0.0290 (6)	0.0316 (6)	0.0351 (6)	0.0014 (4)	−0.0012 (5)	−0.0011 (5)

### Geometric parameters (Å, º)

**Table:**

Cl1—C1	1.7274 (13)	C5—C10	1.4101 (15)
C1—C2	1.3576 (17)	C6—C7	1.3731 (17)
C1—C1^i^	1.428 (3)	C6—H6	0.9300
C2—C3	1.4147 (16)	C7—C8	1.3879 (18)
C2—H2	0.9300	C7—H7	0.9300
C3—N1	1.3564 (14)	C8—C9	1.3719 (18)
C3—C3^i^	1.418 (2)	C8—H8	0.9300
N1—C4	1.3301 (14)	C9—C10	1.4037 (15)
C4—C4^i^	1.438 (2)	C9—H9	0.9300
C4—C5	1.4616 (15)	C10—C10^i^	1.475 (2)
C5—C6	1.3991 (16)		
			
C2—C1—C1^i^	120.17 (8)	C7—C6—C5	120.89 (12)
C2—C1—Cl1	119.62 (11)	C7—C6—H6	119.6
C1^i^—C1—Cl1	120.20 (5)	C5—C6—H6	119.6
C1—C2—C3	120.76 (12)	C6—C7—C8	119.38 (12)
C1—C2—H2	119.6	C6—C7—H7	120.3
C3—C2—H2	119.6	C8—C7—H7	120.3
N1—C3—C2	119.52 (11)	C9—C8—C7	120.69 (12)
N1—C3—C3^i^	121.43 (6)	C9—C8—H8	119.7
C2—C3—C3^i^	119.05 (7)	C7—C8—H8	119.7
C4—N1—C3	116.93 (10)	C8—C9—C10	121.36 (12)
N1—C4—C4^i^	121.64 (6)	C8—C9—H9	119.3
N1—C4—C5	118.57 (10)	C10—C9—H9	119.3
C4^i^—C4—C5	119.79 (6)	C9—C10—C5	117.61 (11)
C6—C5—C10	120.06 (11)	C9—C10—C10^i^	122.27 (7)
C6—C5—C4	119.85 (10)	C5—C10—C10^i^	120.12 (6)
C10—C5—C4	120.08 (10)		
			
C3—N1—C4—C4^i^	−0.40 (17)	N1—C4—C4^i^—N1^i^	0.24 (19)
C4—N1—C3—C2	−179.01 (11)	N1—C4—C4^i^—C5^i^	−179.76 (11)
C4—N1—C3—C3^i^	0.75 (17)	C5—C4—C4^i^—N1^i^	−179.76 (11)
C3—N1—C4—C5	179.59 (11)	C5—C4—C4^i^—C5^i^	0.25 (17)
Cl1—C1—C1^i^—C2^i^	179.08 (10)	C4—C5—C6—C7	−179.86 (12)
C2—C1—C1^i^—C2^i^	−1.40 (19)	C10—C5—C6—C7	−0.32 (18)
Cl1—C1—C1^i^—Cl1^i^	−0.44 (15)	C4—C5—C10—C9	−179.65 (11)
C2—C1—C1^i^—Cl1^i^	179.08 (10)	C4—C5—C10—C10^i^	0.19 (17)
C1^i^—C1—C2—C3	0.67 (18)	C6—C5—C10—C9	0.80 (17)
Cl1—C1—C2—C3	−179.81 (10)	C6—C5—C10—C10^i^	−179.36 (11)
C1—C2—C3—C3^i^	0.74 (18)	C5—C6—C7—C8	−0.3 (2)
C1—C2—C3—N1	−179.50 (12)	C6—C7—C8—C9	0.4 (2)
N1—C3—C3^i^—N1^i^	−0.96 (18)	C7—C8—C9—C10	0.1 (2)
C2—C3—C3^i^—C2^i^	−1.44 (17)	C8—C9—C10—C5	−0.69 (18)
N1—C3—C3^i^—C2^i^	178.80 (11)	C8—C9—C10—C10^i^	179.48 (12)
C2—C3—C3^i^—N1^i^	178.80 (11)	C5—C10—C10^i^—C5^i^	−0.17 (17)
N1—C4—C5—C6	−0.68 (17)	C5—C10—C10^i^—C9^i^	179.66 (11)
N1—C4—C5—C10	179.77 (11)	C9—C10—C10^i^—C5^i^	179.66 (11)
C4^i^—C4—C5—C6	179.32 (11)	C9—C10—C10^i^—C9^i^	−0.51 (18)
C4^i^—C4—C5—C10	−0.24 (17)		

Symmetry code: (i) −*x*, *y*, −*z*+1/2.
